# Faculty’s attitudes and perceptions related to applying motivational principles to their teaching: a mixed methods study

**DOI:** 10.1186/s12909-021-02599-7

**Published:** 2021-03-29

**Authors:** Abigail Grover Snook, Asta B. Schram, Brett D. Jones

**Affiliations:** 1 School of Health Sciences, University of Iceland, 101 Reykjavik, Iceland; 2 School of Education, Virginia Polytechnic Institute and State University, Blacksburg, VA USA

**Keywords:** Motivation, Attitudes, Faculty development, Teacher responsibility, Context, MUSIC model, Motivational strategies, Choices, Feedback

## Abstract

**Background:**

It is uncommon for faculty development professionals to assess faculty attitudes towards their teaching responsibilities and their perceived obstacles to teaching effectiveness. The purposes of this study were (a) to document faculty attitudes and practices related to applying motivation principles, and (b) to identify the perceived contextual factors that may shape these attitudes and practices.

**Methods:**

A sequential explanatory mixed methods design was used. Faculty members (*n* = 272; 32% response rate) were surveyed about their responsibility for and application of the five motivational principles that are part of the MUSIC Model of Motivation: *eMpowerment*, *Usefulness*, *Success*, *Interest*, and *Caring*. Repeated measures ANOVAs and Student’s t-tests were computed to detect differences. Subsequently, two focus groups of faculty members (*n* = 11) interpreted the survey results. We conducted a thematic analysis and used the focus group results to explain the survey results.

**Results:**

Faculty rated their responsibilities for applying principles related to *Usefulness*, *Interest*, and *Caring* significantly higher than they did for *Success* and *eMpowerment*. Most faculty also reported that they actually applied *Usefulness*, *Interest*, and *Caring* strategies within the past year, whereas over half of the faculty applied *Success* strategies and about a third of faculty applied *eMpowerment* strategies. Focus group participants identified factors that affected their ability to apply *eMpowerment* strategies, (e.g., offering choices), including students lacking generic skills (e.g., critical thinking, problem-solving), a lack of confidence in their abilities to implement empowering strategies and meet the needs of students, passive students, and large lecture-type courses. Focus group participants cited obstacles to implementing *Success* strategies (e.g., providing feedback), including difficulty in providing feedback in large courses, lacking time and assistant teachers, limited knowledge of technologies, and lacking skills related to guiding effective student peer feedback.

**Conclusions:**

Faculty appear adequately prepared to implement some types of motivational strategies, but not others, in part due to contextual factors that can influence their attitudes and, ultimately, their application of these strategies. We discuss how these factors affect attitudes and application of motivational strategies and formulate suggestions based on the results.

**Supplementary Information:**

The online version contains supplementary material available at 10.1186/s12909-021-02599-7.

## Introduction

To identify appropriate topics for faculty development, best practices generally call for conducting needs assessments [1, 2]. Authors of a *Best Evidence in Medical Education* (BEME) systematic review on faculty development interventions in the health sciences found that most needs assessments focus on needs related to teaching skills [3]. The authors go on to suggest that faculty development interventions should be re-conceptualized to consider the values of faculty, including opportunities for renewal and reflection on personal and professional attitudes, perceived responsibilities, and practices. Researchers have also increasingly pointed out that faculty development initiatives should focus not only on individual faculty members, but also the contextual factors that might be perceived as obstacles to effective teaching [4]. In sum, this research indicates that there is a demand for needs assessments that consider faculty attitudes and the contexts in which they teach to help identify appropriate faculty development initiatives.

One important topic of interest in faculty development is faculty beliefs and attitudes regarding the use of motivational strategies in teaching. Motivated students engage in activities that help them to learn and excel in academic settings [5]. Because student motivation impacts learning, understanding how to motivate students has received increased interest in health science education in recent years [6]. For example, the *Association for Medical Education in Europe* (AMEE) Guide on motivation in medical education recommends that faculty increase students’ perceptions of content value, self-efficacy, and controllability [7]. In addition to these perceptions, motivation scientists and educational psychologists have also recommended attending to students’ interest and their perceptions of caring [8, 9].

These strategies are summarized and organized with the MUSIC® Model of Motivation [5, 10] (henceforth referred to as the MUSIC Model), which was based on a variety of motivational theories, and designed specifically to help instructors implement motivational strategies into their instruction. MUSIC is an acronym for initial sounds of the five principles of the model (*eMpowerment*, *Usefulness*, *Success, Interest,* and *Caring*) that can be summarized as follows:

*“the instructor needs to ensure that students:* (1) *feel empowered by having the ability to make decisions about some aspects of their learning,* (2) *understand why what they are learning is* ***u****seful for their short- or long-term goals,* (3) *believe that they can succeed if they put forth the effort required,* (4) *are* ***i****nterested in the content and instructional activities, and* (5) *believe that others in the learning environment, such as the instructor and other students, care about their learning and about them as a person”.* [10], (p9)

The MUSIC Model has been validated for use with various student age groups, subject areas, cultures, and languages [11–13], including validation studies conducted with medical students [14] and veterinary medicine students [15].

Although these motivational principles can be taught in faculty development initiatives, faculty may not implement these principles if (a) they do not agree that these principles are their responsibility or (b) they perceive obstacles that hinder their ability to implement them. Identifying faculty’s attitudes and application practices is important because faculty development interventions should target the needs of faculty and it is currently unknown as to what health science faculty needs are related to incorporating motivational principles into their instructional design. For example, do faculty believe that it is their responsibility to consider motivational principles in their instructional design? Do faculty actually incorporate motivational principles into their instructional design? If not, what prevents faculty from incorporating motivational principles into their instructional design?

The specific purposes of this study were (1) to document faculty attitudes and practices related to applying motivational principles to their teaching, and (2) to identify perceived contextual factors that may shape these attitudes and practices. We conducted an explanatory sequential mixed method study to address these purposes, using a quantitative survey to address our first purpose and qualitative focus groups to address both purposes. This was first done with the survey and then by digging deeper for understanding of the quantitative results through focus groups, e.g., understanding how participants conceptualize motivational principles and probing for participants’ possible contextual reasons for their answers [16]. We chose to use the motivational principles provided in the MUSIC Model because the MUSIC Model was designed specifically for instructors to use in educational settings [10], the model is consistent with current, research-based motivational principles, and the model has been used and validated in healthcare professional schools [14, 15]. The specific research questions for this study were:

1. To what extent do health science faculty believe that implementing the motivational principles of the MUSIC Model is a part of their teaching responsibility?
2. To what extent have health science faculty implemented the motivational principles of the MUSIC Model within the last year?
3. Which contextual factors influence faculty members’ implementation of the motivational principles of the MUSIC Model?
4. In what ways could these contextual factors be affecting faculty members’ attitudes and application of motivational strategies?

## Methods

### Setting, population, and context

This study was conducted with faculty of the School of Health Sciences at the University of Iceland during 2017 and 2018. Iceland’s educational system is consistent with the Bologna Process, which seeks to bring coherence to the higher education systems across Europe [17]. The health science programs at this university were between three to six years long in full-time university study, ending with a professional degree and certification to practice as a healthcare professional [18]. Upon graduation, medical students scored similar or higher than students from the United States on the Comprehensive Clinical Skills Examination [19].

The School of Health Sciences consisted of six faculty departments: nursing, pharmacy, food science and nutrition, psychology, odontology, and medicine (which includes physical therapy, biomedical sciences, and radiology). Tenured faculty had both teaching and research responsibilities, usually had a PhD degree, and had oversight of courses that involved sessional/adjunct faculty [20]. Sessional faculty were most often clinicians who taught students directly in the classroom and/or clinic. The university’s 2016–2021 strategy included increasing support of the Centre for Teaching and Learning in an effort to further improve the quality of teaching [21]. Faculty development for the School of Health Sciences was mainly provided by the university’s Centre for Teaching and Learning. Attendance in faculty development was voluntary.

### Ethics

The National BioEthics Committee in Iceland indicated there was no need for them to approve this study given that the data were primarily opinions and contained no sensitive medical data. As per Icelandic regulations, we announced the project to the Icelandic National Data Protection Authority who publicized the project. We followed international regulations with respect to informed participant consent for all aspects of the study (i.e., both the survey and focus group aspects). All methods were performed in accordance with the relevant guidelines and regulations. The researchers had no position of authority over the participants and all participation was voluntary. Participation in the survey served as voluntary consent for the survey part of the study and focus group participants signed consent forms indicating their willingness to participate in the discussions and to have the discussions audiotaped. The study was part of a doctoral project approved by the School of Health Sciences at the University of Iceland.

### Study design

We utilized an explanatory sequential mixed methods study design that included administering a survey followed by conducting focus groups. As described by Creswell and Plano Clark, the first strand included collecting and analysing the quantitative data, subsequently connecting it to the qualitative strand - “the point of interface for mixing – by identifying specific quantitative results that call for additional explanation and using these results to guide the development of the qualitative strand … Finally the researcher interprets to what extent and in what ways the qualitative results explain and add insight into the quantitative results and what overall is learned in response to the study’s purpose” (p. 83) [22].

We designed the study sequentially to first use a quantitative survey to assess faculty attitudes towards and application of motivational principles. The quantitative survey data were collected as a primary source, thus having somewhat more priority over the qualitative strand [22]. Transitioning from the survey data, we followed up by asking the focus group participants about the survey results, to further support or not support these findings, or shed light on the survey responses. This is where the two phases – quantitative and qualitative – connect in the study [23]. We speculated that teachers’ attitudes towards and application of motivational principles might be affected by a variety of factors and gave the focus group participants the permission to discuss freely. We integrated/merged the data by using the focus group results to explain and add insight to the quantitative results in a combined analysis and discussion [22].

As is common in the explanatory design, the philosophical assumptions behind our study began within the postpositivist perspective shifting over to the more constructivist perspective in the qualitative strand [22]. The study was based on pragmatic study design, i.e., the individual decision maker (faculty member) in a real-world context (the classroom) is asked to view a problem in its broadest context (attitudes towards and application of motivational principles) to generate possible solutions to that problem [24]. In other words, reality is driven by experience (ontology); knowledge needs to have utility (epistemology).

### Quantitative phase

#### Participants and procedure

A total of 863 (212 tenured and 651 sessional faculty) email addresses were available through various sources. An email with a link to participate in the survey was sent to all 863 faculty in two waves of data collection in consecutive months (October – November 2017). Faculty were sent up to three reminder emails if they had not yet completed the survey. The study information and the invitations were emailed with a code specific to that email address and a link to the online survey. The codes were used for three purposes: to be able to send reminder emails to non-responders, to distribute renumerations if the participant requested (a $20 gift card), and for purposive sampling in the qualitative phase of the study. It was explained to the participants in the invitation that their voluntary participation in the online survey would serve as informed consent.

We received 272 usable responses (a 32% response rate). In Table 1, we provide a demographic comparison of the total number and distribution of tenured faculty at the School of Health Sciences with tenured faculty within our sample, as well as a comparison of all faculty within our sample [25]. A comparison of the total number of sessional faculty to our sample was not possible because that information was not available. Our sample was similar in distribution within faculties but had proportionately more females and fewer males than the tenured faculty distribution at the School of Health Sciences.

**Table 1:** Participant demographics

	Total TF at SHS *N* = 212	Sample (TF) *n* = 78	Sample (TF + SF) *n* = 272
Medicine faculty	119 (56%)	42 (54%)	158 (58%)
Nursing faculty	32 (15%)	15 (19%)	49 (18%)
Odontology faculty	19 (9%)	5 (6%)	8 (3%)
Other faculty	42 (20%)	16 (21%)	30 (11%)
Female	95 (45%)	40 (51%)	153 (56%)
Male	117 (55%)	30 (38%)	77 (28%)

*TF* Tenured faculty, *SHS* School of Health Sciences, *SF* Sessional faculty, Sample = respondents included in our analysis; Other = Nutrition and Food science, Pharmacy, and Psychology

Of our sample, 61% were sessional faculty and 29% were tenured faculty. Fifty percent of the sample participants were under the age of 52, and 37% were 53 years old or older.

#### Survey items

The 10 survey items used in this study were included as part of a larger survey that also included questions unrelated to the present study. Five items were included to answer Research Question 1 (To what extent do health science faculty believe that implementing the motivational principals of the MUSIC Model is a part of their teaching responsibility?). Each of the five items represented one of the five motivation principles in the MUSIC Model (i.e., eMpowerment, Usefulness, Success, Interest, Caring) [5]. The items were as follows: “It is part of my responsibility as a teacher to...”: (1) “offer students choices in some aspects of their learning” (*eMpowerment*); (2) “explain how the learning process or subject material is useful to student goals” (*Usefulness*); (3) “provide feedback and organization to ensure students’ perception of success” (*Success*); (4) “generate interest in the subject” (*Interest*); and (5) “communicate caring and respect for students (and their goals)” (*Caring*). Items were rated on a 6-point Likert scale (1 = “strongly disagree”, 2 = “disagree”, 3 = “somewhat disagree”, 4 = “somewhat agree”, 5 = “agree”, 6 = “strongly agree”, or “choose not to answer” [no weight]).

Five additional items were included in the survey to answer Research Question 2 (To what extent have health science faculty implemented the motivational principles of the MUSIC Model within the last year?). These items were designed to determine if faculty had applied the MUSIC principles to their teaching within the last year. These items were as follows: “In my last course, I …” : (1) gave my students choices in some aspects of their learning” (*eMpowerment*); (2) “explained to my students why the knowledge they are learning could be useful to their goals” (*Usefulness*); (3) “with good organization and feedback, strengthened my students’ belief that they could be successful” (*Success*); (4) “generated student interest in the material” (*Interest*); and (5) “communicated that I cared for and respected my students” (*Caring*). The same Likert scale as described previously was used to rate the items.

We modelled these 10 attitude and application items to be similar to those on the MUSIC Model of Academic Motivation Inventory [26], which has been shown to be valid for use with college students in the USA [11] and outside the USA [13], and with students in professional schools [14, 15, 27]. To adapt the survey to Icelandic, we utilized guidelines for the cross-cultural adaptation of surveys [28]. We also used wording from the validated translation and adaptation of the middle/high school version of the MUSIC Model Inventory from English to Icelandic [29]. We collected participant information at the end of the survey by asking faculty about the following: their gender, their age range, their teacher type (tenured or sessional), and for which department they primarily worked (see Table 1).

#### Quantitative phase data analysis

We tested the 10 questions with individuals who provided feedback and did not identify any items that were problematic due to the translation process. We calculated frequencies for participants’ levels of agreement with the 10 responsibility and application survey items. We also calculated repeated measures ANOVAs: one to compare responsibility statements to each other and another to compare application statements to each other. Finally, we calculated Student’s paired t-test values to compare differences between each responsibility statement and its corresponding application statement. As expected, the result indicated a need to dig deeper and shed a better light on the survey results, bringing us into the next phase – the qualitative phase.

### Qualitative phase

#### Research team and reflexivity

We used the COREQ checklist for reporting the findings of the qualitative phase [30]. The facilitator of the focus groups had earned a Ph.D., was the director of the Educational Research Institute at the School of Education with extensive experience in leading focus groups, was a female, and was not one of the authors of the study. She was unknown to the focus group participants and in no position of authority over them. She was introduced as knowledgeable about teaching and interested in the topic of motivation, but relatively unfamiliar with healthcare education. The two primary researchers (AGS and ABS) served as assistants and observers on the focus group team. AGS was a doctoral student and teacher at the time, and had experience in qualitative data collection and analysis [31]. ABS had earned a Ph.D. and was a faculty developer for the School of Health Sciences; she had experience in qualitative data collection and analysis [31]. Neither ABS nor AGS were known to any considerable extent by the focus group participants. The participants knew that the focus groups were part of AGS’s doctoral project and that ABS was her advisor. Both ABS and AGS introduced themselves as having interest in faculty development and in finding ways to support teachers in their teaching.

#### Qualitative study design

We used a constructivist approach as we sought to understand individual experiences of applying motivational strategies and identify patterns in these subjective experiences [22]. We chose focus groups as our method because it allowed us to listen to participants discuss freely the overall survey responses and to observe how they interacted with one another in discussing the material [32]. We used a sample of convenience within a purposive sampling of the 272 teachers who had participated in the survey. Using this method allowed us to better connect our quantitative and qualitative results [23]. Participants were contacted through email and invited to participate. Eleven teachers volunteered to participate in the focus groups, and none refused to participate nor dropped out during the study.

The study took place after work hours in one of the Health Science School buildings. There were no other people present other than the participants and the research team. We formed two focus groups (Group 1, *n* = 6; Group 2, *n* = 5) as small groups (4 to 6) are known to affect communication in a positive way [33]. The groups were formed to mix faculty from different disciplines (medicine, *n* = 4; nursing, *n* = 2; physical therapy, *n* = 4; and nutrition, *n* = 1), types (8 tenure-track, 3 sessional/adjunct), genders (3 males, 8 females), and age ranges (7 ≤ 52 years, 4 ≥ 53 years).

As a way to connect our quantitative and qualitative results [23], we provided the facilitator with the interview guide that included questions based on the summary of the faculty responses to the survey items (the interview guide is provided in the Additional file 1). The interview guide was not pilot-tested nor were the focus groups repeated. The focus group participants signed consent forms, acknowledging that they participated voluntarily and agreeing that the proceedings could be audio-recorded and de-personalized before being presented in any form. The audio recordings were transcribed with the inclusion of vocal inflections (e.g., hesitations, silence, laughter) by a professional transcribing service and were not made available to participants for corrections. The observers (AGS and ABS) took field notes and also participated at times in the discussion. The focus groups met in November of 2018 and each group lasted 1.5 h. Saturation was considered and determined by the repeating of themes between the two focus groups.

#### Qualitative phase data analysis

We chose an inductive thematic analysis approach [34] and utilized no software in the analysis. We followed the six phases of thematic analysis suggested by Braun and Clarke [35]. After immersion in the data, we generated initial codes based on the main thought(s) expressed when each participant spoke, while color-coding the response to which motivational strategy it addressed. AGS was the primary coder for the transcriptions with ABS checking the AGS codes and AGS and ABS discussing any disagreements and settling them by mutual consent. There was a special emphasis on the motivational strategies that teachers felt less responsible for, which became the main themes. Codes were then grouped together based on similar thoughts and became the subthemes under each theme, with some subthemes pertaining to multiple themes. No feedback was gathered from participants regarding themes and subthemes generated. After determining that the thematic map captured our data, we defined our themes [22, 35]. Together, AGS and ABS identified representative quotations for each of the subthemes and these were translated and back-translated from Icelandic to English by independent bilingual experts according to guidelines [36].

## Results

### Quantitative phase

**Fig. 1:**
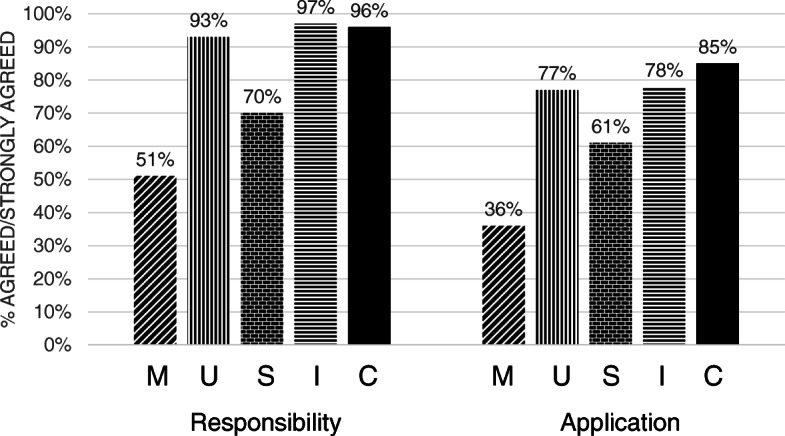
Agreement/strong agreement with MUSIC Model principles. Responsibility = “It is part of my responsibility as a teacher to...”; Application = “Consider your teaching in the last year and evaluate the following statements: I...” M = give students choices in some aspects of their learning; U = explain to students why the knowledge and skills they are learning could be useful to their goals; S = strengthen, by good organization and feedback, my students belief that they can succeed; I = generate student interest about my subject matter; C = communicate respect and caring to my students

Faculty attitudes about their responsibilities for implementing each of the five MUSIC principles and about their actual application of each motivational principle is provided in Fig. 1. Faculty rated their responsibilities for applying principles related to *Interest* and *Caring* the highest (96% or higher agreement/strong agreement), followed by *Usefulness* (93%), *Success* (70%) and *eMpowerment* (51%) (as shown on left side of Fig. 1). A repeated measures ANOVA indicated significant differences (*p* < .001) for all responsibility statements except no difference was detected between *Interest* and *Caring*. Consistent with this pattern, 77% or more of faculty reported that they actually applied *Usefulness*, *Interest*, and *Caring* strategies within the past year, whereas 61% of faculty provided good organization or feedback to foster students’ success (*Success*) and 36% of faculty reported offering choices (*eMpowerment*) (as shown on the right side of Fig. 1). A repeated measures ANOVA indicated significant differences (*p* < .001) between all application statements except no differences were detected between *Interest*, *Usefulness*, and *Caring*. Student’s paired t-test values also indicated significantly lower values (*p* < .001) for each application item when compared to the corresponding responsibility item. For example, although 97% of faculty agreed it was their responsibility to generate interest in the subject, significantly fewer (78%) actually reported generating student interest in the material during their last course. The results in Fig. 1 were utilized to develop the interview guide for the qualitative study as part of the mixing of the results.

### Qualitative phase

The focus group participants discussed and confirmed the findings of the survey: they felt responsible for the *Usefulness, Interest,* and *Caring* principles of the MUSIC model and indicated that they did not feel they needed further support by administrators or others to implement these principles. When asked why they thought the survey results showed that teachers felt less prepared or supported to implement the *eMpowerment* and *Success* principles, the participants indicated that they did feel responsible for the strategies of offering choices (*eMpowerment*) and providing feedback (*Success*) to some degree. However, they did not take full responsibility for them because of contextual factors that were hindering their implementation.

The themes identified from the thematic analysis were the following: (1) Responsibilities as a teacher; (2) Factors making it difficult to offer choices (*eMpowerment*); and (3) Factors making it difficult to provide feedback (*Success*). The themes, subthemes, and representative quotations identified during the focus groups are provided in Table 2. Faculty identified several obstacles that prevented them from fully offering choices: their lack of confidence in their ability to offer choices and meet students’ needs; having to teach large, fixed-content courses; not having mentors; not having stories of how other faculty have successfully implemented choice strategies; and students’ lack of ability to problem solve, critically think, and be active learners. The subthemes also identified why it was difficult for them to provide feedback as a way to promote student success: they lacked the time, they had to teach large courses, they did not have assistant teachers, they did not know which technology would be most effective in helping them to provide feedback, and when encouraged to provide various types of feedback (e.g., student peer feedback), they did not know how to effectively guide students to provide peer feedback in group work.

**Table 2:** Focus Group Results

Theme	Subtheme	Quotes from participants that support subtheme
Responsibilities as a teacher	I am responsible for *Caring* and *Usefulness*	I really like this Caring result (FG2, R2)
		No one else can do that (Caring) so that answer (that I am responsible) seemed appropriate (FG2, R2)
		(You need) to explain why they are doing a project, why they are working together as group, you (need) to explain (with emphasis) everything (FG1, R1)
	I am not entirely responsible for *Success* and *eMpowerment*	because I cannot be quite responsible for these (eMpowerment and Success) myself, although I wanted to do it (FG2, R2)
	I am responsible for providing feedback (*Success*)	(I am) absolutely responsible for that (providing feedback) but the framework does not allow for it (FG1, R4)
	I am aware that choices engage students (*eMpowerment*)	material that they are interested in … their own material, they are excited about it, I don´t decide what they should do, it works better that way (FG1, R5)
Factors making it difficult to offer choices *(eMpowerment)*	I lack confidence in my ability to offer choices and meet students' needs	We are not accustomed to (other teaching methods), we were all taught that someone came in, he came, and he left, (implied lecture format)…. and that was our university education (FG1, R2)
		Then we came back (as educators) and then the demands were different, different thoughts, and it just takes time (to learn them) (FG2, R1)
		(You need to) just make the decision (FG2, R1)
	Institutional issues of large, fixed content courses	Large, basic science courses are challenging (many participants)
		Some of the courses are very much, set in stone (rigid in form and organization) (FG1, R6)
	I do not have access to mentors and success stories	I need to learn that (FG2, R4) (in response to stories told by other participants about offering choices and problem-based learning)
		(I need) to get somebody to be with me, to guide me, someone who knows what to do and has done this before (FG1, R1)
	I am concerned that students lack generic skills and take a passive role towards learning	They (the students) are not as able, as I would have thought, to search, find solutions (FG2, R2)
		(I am not sure) that they really have learned to gather knowledge through critical thinking (FG1, R1)
		Can the student find out (the solution), can the student uncover the reasons behind it (the problem)? (FG2, R1)
		There were about half of them that found flipped teaching to be awesome, but those who could not be bothered to prepare themselves found it terribly (with emphasis) difficult (FG2, R1)
Factors making it difficult to provide feedback *(Success)*	Institutional factors of not enough time, large classes, no assistant teachers	The (lack of) feedback it is just completely not any more complicated than just a horrible lack of time (FG2, R2)
		I know that the students get much (with emphasis) more out of this when I provide feedback and they can turn it in again, but I just can’t always do that (FG2, R4)
		(A class of) 10-15 people – giving feedback has gone very well with that number (FG1, R3)
		Facilitator: You mean that assistance with a large class should be automatic? Participant: If you want good feedback, that is what it takes. (FG2, R1)
	I do not know how to effectively guide students in providing student peer feedback in group work	Some just say (that) everybody did fine, and it´s all good, if it´s just because it is obviously difficult to say that the friend is not doing his part… but then there are others who are completely not shy, so it varies a bit. (FG2, R5)
		(Teacher shared successful peer review story and then asked if students were willing) they are very (with emphasis) willing, they find it extremely enjoyable (FG2, R5)
	I do not know about technology that could help provide feedback	Did not know about technology that provides feedback (many)

## Discussion

**Fig. 2:**
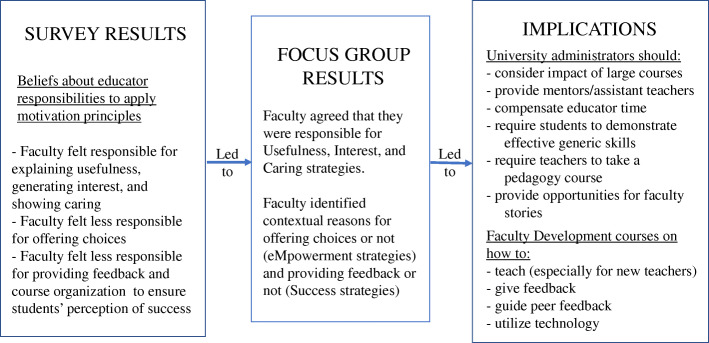
Suggestions for improvement based on the survey and focus group results

The two purposes of this study were (1) to document faculty attitudes and practices related to applying motivation principles to their teaching and (2) to identify the perceived contextual factors that may shape these attitudes and practices. We were able to establish through the survey – supported by the focus group responses – that faculty felt significantly less responsible for offering choices to students *(eMpowerment)* and for using good organization and feedback to help students believe that they can succeed in course activities *(Success)*. We also documented from the survey results that they perceived they were using these *eMpowerment* and *Success* principles less in the classroom and reported significant gaps between what they perceived to be their responsibility and the extent to which they actually applied the five MUSIC Model principles. This gap suggested that there were obstacles hindering faculty from applying these principles. Through the focus groups, we were able to identify some of the contextual factors specific to the *eMpowerment* and *Success* principles. This allowed us to elaborate on how these factors might be affecting their attitudes and actions. Possible implications for university administrators and faculty developers related to the discussion points provided in this section are summarized in Fig. 2. These implications will be discussed in the following sections.

### Faculty’s perceived responsibilities

The survey results indicated that almost all faculty agreed that motivation principles related to *Interest*, *Caring*, and *Usefulness* were their responsibility (see Fig. 1). In other words, they felt responsible for triggering students’ interest, caring about students’ learning and well-being, and explaining why learning the content and skills are useful for the students’ goals and future. The focus group results supported these results – that participants took full responsibility for communicating *Caring* to their students, generating *Interest* in the subject material, and acknowledged that explaining the *Usefulness* of gaining knowledge and skills is important (Table 2). The AMEE Guide on motivation in medical education [7] also notes the importance of perceived value, which is similar to (if not the same as) the combined principles of *Interest* and *Usefulness*. However, the *Caring* principle for students is not explicitly highlighted in the AMEE Guide. Given the importance of caring to students’ motivation in educational settings [9], caring relationships (between the students and the faculty member and between students) is an area that could be more explicitly explored in health science research.

Many faculty also agreed that the *eMpowerment* and *Success* principles were their responsibility (51 and 70%, respectively); however, these principles were rated significantly lower than the other principles. Empowering students by offering choices contributes to perceived controllability and is a central component in control, self-determination, and interest theories [37–39]. Promoting students’ *Success* by giving constructive feedback on a regular basis contributes to self-efficacy because it informs students about their competence and gives them an opportunity to assess whether their learning strategies are working. The importance of perceived controllability and self-efficacy is also noted in the AMEE Guide [7].

When it comes to actually applying motivational principles, significantly fewer faculty in our study reported using *eMpowerment* and *Success* principles in their teaching, as compared to using *Usefulness*, *Interest*, and *Caring* principles. Focus group participants understood the importance and effectiveness of using choices as a form of empowering students and felt that providing feedback to promote students’ success was their responsibility to some degree (Table 2). However, the participants described factors that hindered their application of these strategies. In the following sections, we discuss the factors affecting attitudes and application of *eMpowerment* and *Success* principles because most focus group comments were directly related to these principles. In doing so, we use the qualitative focus group results to explain the attitudes and application trends identified in the quantitative survey results.

### Empowerment principles

One reason the focus group participants mentioned for not offering choices as a form of empowerment was that they were unsure if their students had the necessary critical thinking skills to manage the increased responsibility and critical thinking that accompany increased choices. Critical thinking, problem solving, and reasoning skills are often labelled as “generic skills” (i.e., skills that may be applied to a range of different situations) and, within the context of healthcare, these skills are essential to success and achievement [40, 41]. Other researchers have also questioned whether healthcare students have these skills [41] and whether these skills are being developed during their healthcare education [42, 43].

Two other reasons the focus group participants provided for not offering choices were related to their lack of confidence in their own abilities. First, they felt more comfortable teaching the same way they were taught, which was in lecture format. These findings are consistent with other studies that have found that faculty practices were impacted by what was modelled to them in the past [44]. Second, the teachers did not believe they could meet the needs of today’s students. Pettit et al. [45] found that medical students prefer a variety of teaching methods and value choice, flexibility, efficiency, and the ability to control the pace of their learning. These issues could be addressed through empowering students by offering choices [10]. However, without preparation for the role of teaching through faculty development, DaRosa et al. [46] concluded that faculty will lack the confidence to use unfamiliar teaching techniques. The focus group findings in our study also indicated that participants wanted help from trained mentors, which is something that has been shown to help increase faculty confidence [47]. Just as students may be de-motivated by not feeling they can succeed in a course [5], faculty may be de-motivated by not feeling they can succeed at offering choices.

Another way to increase faculty confidence may be to create the time and the platform for them to share their successes and struggles in implementing empowerment strategies. When participants in our study heard stories about teaching from the other participants during the focus group meetings, they seemed to reflect on their own teaching, an important first step when assessing attitudes and practices [10]. Other researchers have also found that sharing stories can help faculty reflect on their own teaching [48]. The re-telling of successful teaching stories where choices are offered may impact both a teachers’ attitude towards offering choices and also give them practical suggestions for implementation, which may affect their application of offering choices.

The fact that some students take a passive role towards learning may also limit faculty’s use of empowering strategies. Love et al. [49] identified student passivity as an obstacle for medical faculty who want to improve their teaching, similar to what our participants experienced when using flipped teaching. A possible solution is to gradually introduce students to more active teaching techniques [50], as a way to move students towards more independent learning. Sierens et al. [51] demonstrated that structure (in the form of help, instructions, and clear expectations) was required if the goal was to predict self-regulated learning. Helping a student to be more self-regulated and an active learner can be considered another form of empowering students because it leads to greater student autonomy. If faculty perceive that their students are more active in their learning, they may consider the option of offering choices.

Large lecture-type courses were also mentioned as obstacles to offering choices. Teaching large lecture-style courses can be difficult for faculty, especially those faculty with a limited pedagogical background. Large, fixed-content courses may make teachers feel they have no options (or autonomy) to offer choices, and thus, negatively affect faculty’s attitudes about their responsibility to apply empowerment strategies.

### Success principles

Large, lecture-type courses were also named as obstacles to providing feedback because of the large amount of time involved in providing feedback to so many students. One solution mentioned by the focus group participants for the *Success* principle was for administrators to provide faculty with assistant teachers to help them provide regular feedback and grade assignments. This finding is consistent with other researchers who have noted that faculty often lack the time needed to provide quality feedback and assessment; and therefore, could use help in doing so [49]. If faculty perceive that they are being asked to provide effective feedback to students but are not being supported by paid time or assistant teachers [49, 52, 53], they may believe that it is not a reasonable expectation to provide regular feedback and, in turn, provide less feedback.

Many of our focus group participants indicated that they had been encouraged to try to use student peer feedback as a way to provide feedback to students. However, they seemed to struggle with how to guide students to provide effective feedback to their peers. Nofziger et al. [54] suggested that students need to receive training from faculty in how to give specific, constructive feedback to each other. In addition, the focus group participants seemed unfamiliar with technologies that might assist them in providing feedback. Love et al. [49] reported that being able to use technology in teaching was identified as one of main drivers behind faculty’s motivation to want to improve their teaching. Given that our focus group participants appeared supportive of the idea of student peer feedback and using technology to do so, the reasons that they did not use these strategies were related to a lack of training in how to guide students to provide effective peer feedback and in how to use technology to provide feedback, in general.

### Limitations and future research

In this section, we list a few limitations of our study along with possibilities for future research. First, the study was conducted at one health science school, which may limit the generalizability of the results. Nonetheless, many of our themes were consistent with other research findings and we believe that the participating university represents a fairly typical health sciences school that is trying to improve its faculty development and teaching. Future studies could include a wider variety of health science schools from different countries. Second, the use of only two focus groups may have limited our ability to capture all of the main themes [55]. Interviewing more focus groups could provide other perspectives that were not identified in the present study.

Third, we used the MUSIC Model of Motivation as a framework to organize faculty perceptions about motivation-related design principles. We are aware of other theories that cover similar constructs, such as self-determination theory and expectancy-value theory, but we chose to use the MUSIC Model. The MUSIC Model allowed us to take a multidimensional approach to examining perceptions that are most closely related to student motivation. However, because we only asked one survey item per MUSIC Model component, we only captured part of each larger MUSIC Model principle. For example, the application item related to the *Success* principle focused on how organization and feedback affected students’ success beliefs, but it did not ask about other instructional strategies that can affect students’ success beliefs (e.g., adjusting the difficulty levels of assignments). Future research could provide survey items related to a broader range of strategies that faculty could use to affect students’ perceptions of each of the MUSIC Model principles.

Other suggestions for future research include conducting further studies related to examining the importance of *Caring* to health science students. Also, researchers could test the interventions and implications we suggested for their effectiveness. It would be particularly useful to determine whether these suggested strategies lead to increased student motivation and then to increased student learning and achievement.

## Conclusions

Our study adds to the literature by asking faculty about their attitudes and identifying perceived factors that affect their attitudes about and application of motivational strategies. By exploring contextual factors that affect teaching attitudes and practices, our qualitative results connected to and helped explain our quantitative results [3, 4], leading to a greater understanding of the challenges faculty face when applying motivational principles. Interestingly, many of the factors named as hindrances to faculty’s application of *eMpowerment* principles also appeared to negatively affect their attitudes towards strategies related to those principles. Considering and addressing factors that influence or shape faculty attitudes towards their responsibilities as teachers may help faculty development professionals develop effective interventions that increase the use of motivational strategies in the classroom.

## Supplementary Information

**Additional file 1.**

## Data Availability

The datasets used and/or analysed during the current study are available from the corresponding author on reasonable request.

## Abbreviations

ABS: Asta Bryndis Schram
AGS: Abigail Grover Snook
AMEE: Association for Medical Education in Europe
BEME: Best Evidence in Medical Education
CBL: Case-based learning
MUSIC Model: MUSIC Model of Motivation
