# Supplementary material for: Faculty’s attitudes and perceptions related to applying motivational principles to their teaching: a mixed methods study
Source: BMC Med Educ. 2021 Mar 29;21:188. doi: 10.1186/s12909-021-02599-7 (PMC8008516; doi:10.1186/s12909-021-02599-7)
Supplement: Supplementary file 1 — Additional file 1. [file 12909_2021_2599_MOESM1_ESM.docx]

Interview Guide (primarily to trigger conversation)

1. Explain and have participants sign consent forms. Start recordings.
2. Introductions – everyone introduces themselves
   1. Department, teach undergraduate or graduate students, subject taught
3. Introduce results using handout with graph of responsibilities (left half of Fig. 1) and application (right half of Fig. 1)
   1. Have participants read over motivational statements
   2. Point out that U, I, and C are high for responsibility and application. Point out that M and S are lower for responsibility and application.
4. Discussion
   1. Any thoughts on the results in general? Any of the motivational statements unclear?
   2. Any thoughts on the high results for U, I, and C? Why do you think teachers thought these were their responsibilities?
   3. Choices
      1. Do you feel that offering choices empowers students or do you associate empowering students with other ideas?
      2. Do you think that offering choices is your responsibility? Why or why not?
      3. Do you offer your students choices? Why or why not?
      4. What helps you and what hinders you from offering some choices to your students in the classroom?
   4. Feedback
      1. Do your think that providing feedback is your responsibility? Why or why not?
      2. Do you provide adequate feedback in your classroom? Why or why not?
      3. What helps you and what hinders you from providing feedback?
         1. In your experience, do you think that peer feedback works? Share some of your experiences with group work and peer feedback
   5. Have you had experiences trying to change your teaching? Would you share?
   6. Any more thoughts on the results of the survey?
5. Thank you
